# A Highly Conserved Poc1 Protein Characterized in Embryos of the Hydrozoan *Clytia hemisphaerica:* Localization and Functional Studies

**DOI:** 10.1371/journal.pone.0013994

**Published:** 2010-11-16

**Authors:** Cécile Fourrage, Sandra Chevalier, Evelyn Houliston

**Affiliations:** Université Pierre et Marie Curie and CNRS, Developmental Biology Unit, Villefranche-sur-Mer, France; Virginia Tech, United States of America

## Abstract

Poc1 (Protein of Centriole 1) proteins are highly conserved WD40 domain-containing centriole components, well characterized in the alga *Chlamydomonas,* the ciliated protazoan *Tetrahymena,* the insect *Drosophila* and in vertebrate cells including *Xenopus* and zebrafish embryos. Functions and localizations related to the centriole and ciliary axoneme have been demonstrated for Poc1 in a range of species. The vertebrate Poc1 protein has also been reported to show an additional association with mitochondria, including enrichment in the specialized “germ plasm” region of *Xenopus* oocytes. We have identified and characterized a highly conserved Poc1 protein in the cnidarian *Clytia hemisphaerica. Clytia* Poc1 mRNA was found to be strongly expressed in eggs and early embryos, showing a punctate perinuclear localization in young oocytes. Fluorescence-tagged Poc1 proteins expressed in developing embryos showed strong localization to centrioles, including basal bodies. Anti-human Poc1 antibodies decorated mitochondria in *Clytia*, as reported in human cells, but failed to recognise endogenous or fluorescent-tagged *Clytia* Poc1. Injection of specific morpholino oligonucleotides into *Clytia* eggs prior to fertilization to repress Poc1 mRNA translation interfered with cell division from the blastula stage, likely corresponding to when neosynthesis normally takes over from maternally supplied protein. Cell cycle lengthening and arrest were observed, phenotypes consistent with an impaired centriolar biogenesis or function. The specificity of the defects could be demonstrated by injection of synthetic Poc1 mRNA, which restored normal development. We conclude that in *Clytia* embryos, Poc1 has an essentially centriolar localization and function.

## Introduction

Centrioles are intruiging cellular organelles that have fascinated biologists for over a century. They are present in a wide range of eukaryotes including the vast majority of animal cells, and appear to act as organizers not only for the mitotic spindle but also for the interphase microtubule cytoskeleton, thereby playing key role in the positioning and traffic of intracellular organelles. There is thus intense interest in characteristing the structure and composition of the centriole, and in understanding how it interacts with other cellular components.

The WD40 repeat-containing protein Poc1 is an integral component of the centriole and is required for basal body stability and cilia formation. It has been exceptionally well conserved through eukaryotic evolution, being identified in most species in a wide genome survey [1], and has been consistently identified in centriole proteomics studies, for instance basal bodies of the unicellular alga *Chlamydomonas* [2], the ciliated protozoan *Tetrahymena* [3], human centrioles [4] and the mouse sensory cilium complex [5]. Within the centriole, Poc1 shows a precise localization to the microtubule cylinder wall and the proximal/basal cartwheel structure, and is a very early marker for proximal centriole and basal body assembly [3], [6], [7].

Analysis of the cellular distribution of human and *Xenopus* Poc1 proteins has suggested that they may have additional localizations outside the centriole/axoneme. In human cells Poc1a and Poc1b ( =  Pix2 and Pix1 in *Xenopus*) were detected in association not only with centrioles and microtubules but also with mitochondria, while in *Xenopus* eggs Poc1 proteins were detected in a particular region in the oocyte rich in mitochondria known as germ plasm [8]. Germ plasm is a characteristic component of many animal oocytes, often rich in mitochondria, and thought to derive from the vestiges of the oocyte centrosome during oogenesis and to direct germ line development in the cells that inherit it [9].

Studies in *Tetrahymena*, *Chlamydomonas, Drosophila*, human cells and zebrafish embryos have demonstrated a variety of roles for Poc1 proteins relating to their centriole, basal body and axonemal localizations, including in centriole biogenesis and ciliogenesis [7]. Furthermore, a phylogenetic survey showed a systematic absence of Poc1 proteins in species which lack beating cilia, including nematodes, flowering plants and most fungi, supporting the idea of an ancestral role of Poc1 in the growth or activity of motile cilia or flagella [1]. As well as ciliation defects, RNAi depletion of Poc1 in cultured human cells caused a reduction in centriole number in an over-duplication assay, whereas over-expression induced aberrant elongation of the centriolar central microtubule structure [6], and antibody injection disrupted cell division [8]. These phenotypes may reflect a direct role in centriole duplication or secondary effects on reduced centriole stability [8], [10].

We have analyzed Poc1 localization and function during embryonic development in the recently developed experimental model *Clytia hemisphaerica*. *Clytia* belongs to the basally branching animal phylum Cnidaria, and might thus offer evolutionary insights as well as a tractable experimental system [11], the Poc1 sequences in Cnidaria showing strong similarity with those of other studied species [1]. Our results indicate a conserved localization and role for Poc1 in the centriole in *Clytia*, with fluorescence-tagged Poc1 proteins proving to be an exceptional in vivo marker for centrioles/ciliary basal bodies.

## Results

### A Highly Conserved Poc1 Protein in *Clytia*

**Figure 1:**
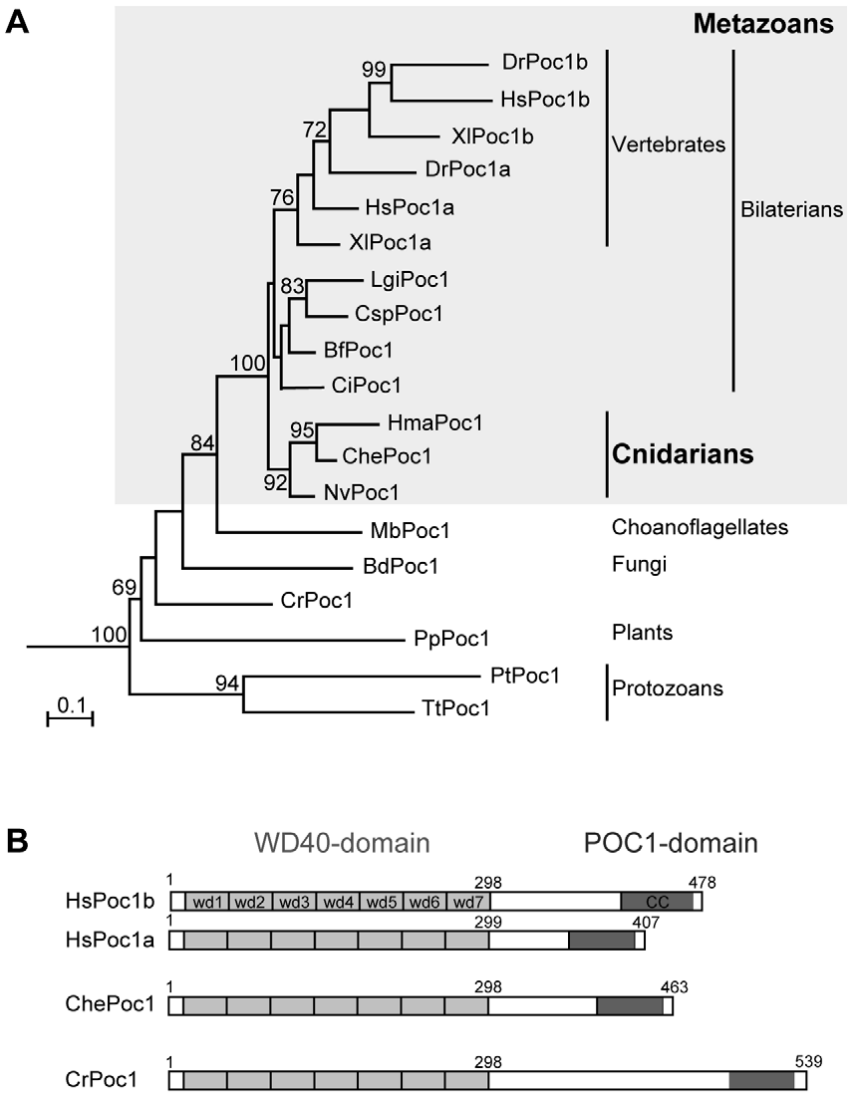
A highly conserved Poc1 in *Clytia*. A. Phylogenetic relationships between eukaryotic Poc1 sequences, deduced by Maximum likelihood (see Materials and Methods). Sequences from the closely related WD40-repeat containing Lis1 proteins, present in the same range of taxa, were included as an out-group. Bootstrap percentages (500 replicates) over 50% are shown. Bd  =  *Batrachochytrium dendrobatidis*; Bf  =  *Branchiostoma floridae*; Ci  =  *Ciona intestinalis*; Che  =  *Clytia hemisphaerica*; Cr  =  *Chlamydomonas reinhardtii*; Csp  =  *Capitella species*; Dr  =  *Dario rerio*; Hma  =  *Hydra magnipapillata*; Hs  =  *Homo sapiens*; Lg  =  *Lottia gigantean*; Mb  =  *Monosiga brevicollis*; Nv  =  *Nematostella vectensis*; Pp  =  *Physcomitrella patens*; Pt  =  *Paramecium tetraurelia*; Tt  =  *Tetrahymena thermophila*; Xl  =  *Xenopus laevis*. Note that the POC1 gene probably underwent duplication in the vertebrate lineage, with the Poc1a sequence subsequently retaining higher similarity to the ancestral protein. Scale: amino acid substitutions per site. B. Schematic comparison of the ChePoc1 sequence with those of human and *Chlamydomonas*. All Poc1 proteins contain a characteristic N-terminal domain comprising 7 WD40 repeats and a C-terminal coiled-coil region containing the POC1 domain.

We isolated a cDNA clone coding for a typical Poc1 family protein designated ChePoc1, following identification by BLAST from a *Clytia* EST collection (see Materials and Methods), and confirmation of its orthology with Poc1 from other species by Phylogenetic analysis (Figure 1A; alignments in Figure S1). ChePoc1 is typical of the Poc1 proteins in containing a N-terminal domain of seven WD40 repeats [1], and a C-terminal coiled-coil region containing a characteristic “POC1” domain (Figure 1B). The WD40 domain, sufficient for centriolar targeting [6], [7], is exceptionally highly conserved across species, whereas the coiled-coil domain is more variable in sequence.

### Poc1 Expression in *Clytia*

**Figure 2:**
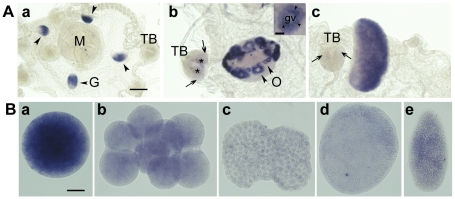
Expression of *Clytia* Poc1. A. Poc1 mRNA detected by in situ hybridization in adult *Clytia* medusae. a: Immature female. b: Mature female gonad showing high Poc1 mRNA concentrations in small and medium sized oocytes (inset shows an individual oocyte at higher magnification, with black arrowheads indicating the particulate distribution of the probe around the oocyte nucleus. Scale bar: 50 µm). c: Mature male gonad. Poc1 is expressed strongly in male and female gonads, and weakly at the base of the tentacle bulb (arrows in b and c). G  =  gonad; M =  manubrium; TB  =  tentacle bulb; O  =  oocytes; gv  =  germinal vesicle. Asterisks in b indicate non-specific staining of the tentacle bulb endoderm, frequently observed in *Clytia* in situs [1]. Scale bar: 100 µm. B. Poc1 mRNA detected by in situ hybridization of eggs, embryos and larvae. Poc1 maternal mRNA is detected strongly in the unfertilized egg (a) but decreases progressively through cleavage (b: 8-cell stage) and blastula (c) stages. The relatively higher signal in gastrula (d) and planula (e) stages probably reflects new trancription starting from the blastula stage. Scale bar: 50 µm.

In situ hybridization analysis of adult *Clytia* medusae (Figure 2A) revealed that as in *Xenopus* [8], Poc1 mRNA is predominantly expressed in the gonads with only low expression detectable in other tissues (including at the base of the tentacle bulbs, a site of intense stem cell division [12]). In female gonads (Figure 2Ab), ChePoc1 mRNA was detected in oocytes at all stages of oogenesis. Typically for maternally expressed RNAs, it was strongly concentrated in the small previtellogenic stage I and II oocytes positioned closest to the medusa bell [13], which are arrayed as two lines when the medusa is viewed through the bell as in Figure 2Ab. In larger, more distally-positioned later-stage oocytes, positioned behind the previtellogenic rows in this image, maternally expressed mRNAs such as Poc1 are diluted by yolk accumulation [13]. Within the oocyte, Poc1 mRNA appeared concentrated in a patchy distribution around the nucleus ( = germinal vesicle) (Figure 2Ab inset), suggesting an association with perinuclear organelles such as mitochondria (see below), as noted also for a set of germ-plasm marker gene mRNAs (Leclère et al, unpublished data). In male gonads, Poc1 mRNA was detected in the developing spermatocytes, which are positioned in the most peripheral zone of the gonad [14], [15].

In situ hybridization of progressive embryonic stages (Figure 2B) indicated that ChePoc1 mRNA is highly concentrated in unfertilized eggs, with the signal appearing to decrease through the period of cleavage division until the blastula stage, presumably reflecting progressive exhaustion of the maternal mRNA stock. The transition from maternal to zygotic gene expression in *Clytia* is thought to occur at around the mid-blastula stage [16]. In all embryonic stages Poc1 mRNA was detected uniformly, with no evidence for preferential expression in any cell type.

These In situ hybridization observations indicate that Poc1 mRNA is stockpiled in the egg to contribute to the maternal phase of embryogenesis, and shows elevated expression in zones of cell proliferation (male gonads and tentacle stem cells), but is otherwise present at low levels across cell types in the larva (‘planula’) and adult jellyfish.

### *Clytia* Poc1 Localizes to Centrioles and Basal Bodies

**Figure 3:**
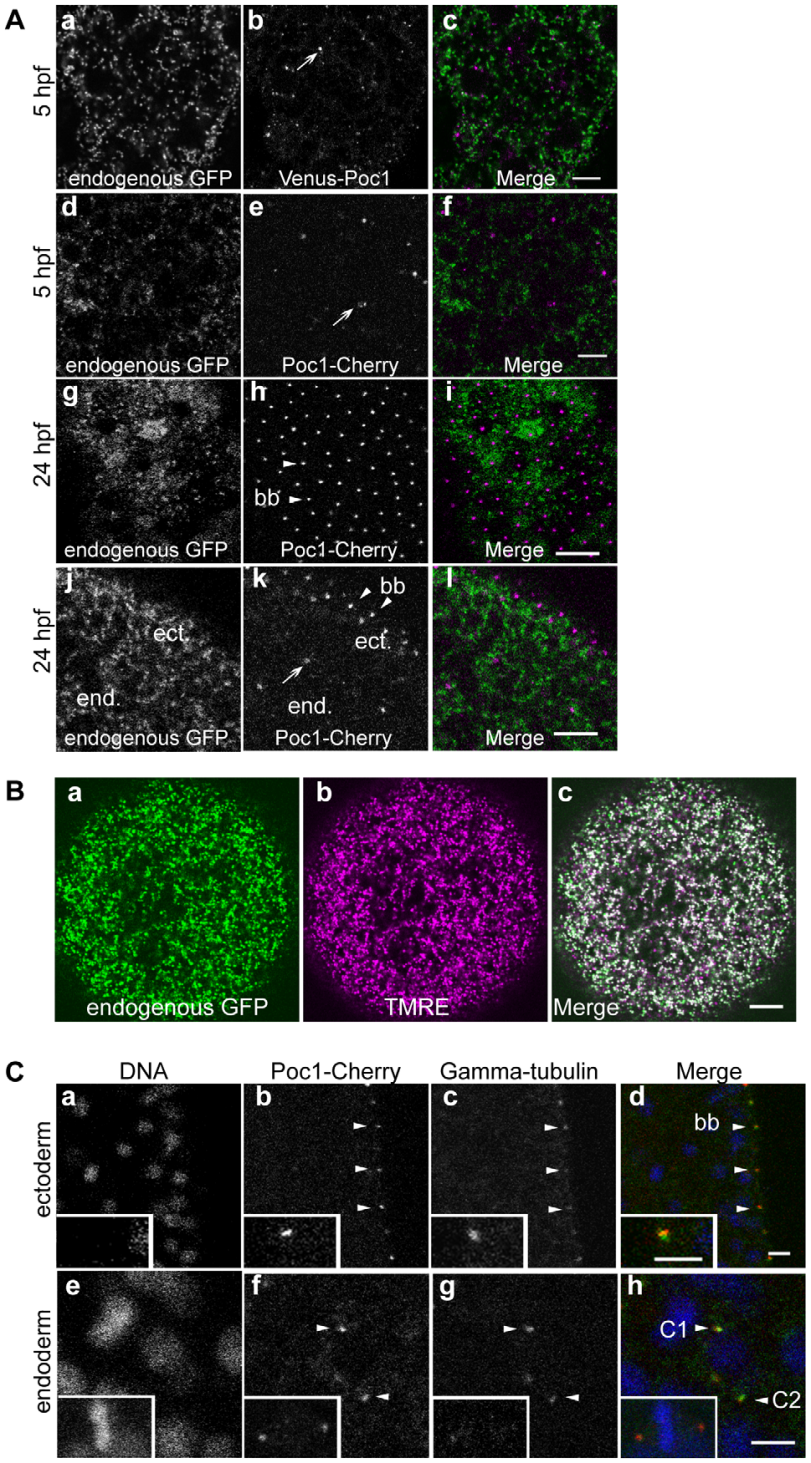
*Clytia* Poc1 is targeted to centrioles. Confocal images of embryos expressing fluorescent–tagged ChePoc1 protein following microinjection of mRNA constructs into the egg prior to fertilization. A. blastulae fixed at 5hpf (a–f) and planula larvae fixed at 24hpf (g,i: plane through apical ectoderm; j,k,l: plane through both endoderm (end) and ectoderm (ect)). Both fluorescent Poc1 proteins clearly localize to punctate aggregates positioned on the apical side of each ciliated ectoderm cell of the larva (bb/arrowheads: basal bodies), and randomly in blastula cells and larval endoderm (arrows). a,d,g,j: endogenous GFP showing the distribution of mitochondria in the cytoplasm of each cell (see B); b: Venus-Poc1; e,h,k: Poc1-mCherry; c,f,i,j: merged images. Scale bar: 10 µm. B. Confocal images of an unfertilized egg demonstrating that maternal endogenous *Clytia* GFP (a) is a natural mitochondrial marker, colocalizing with the mitochondrial dye TMRE (b) as seen in the merged image (c). C. Confocal images of larval cells expressing Poc1-mCherry following micro-injection of mRNA constructs into the egg prior to fertilization, fixed, and then processed for immunofluorescence with anti-gamma tubulin to locate centrosomes. Poc1-mCherry colocalizes with gamma-tubulin at the position of the basal body (bb/arrowheads- enlarged in insert) in ectoderm cells (a,b,c,d) and at the centrosomes of dividing cells in the endoderm (e,f,g,h) (C1/C2 =  centrosomes close to prometaphase chromatin; metaphase cell in inset). a,e: DNA (Hoechst); b,f: Poc1-Cherry; c,g: gamma tubulin; d,h: merge (blue = DNA; red = Poc1-Cherry; green = gamma tubulin). Scale bars: 5 µm.

To study localization of the *Clytia* Poc1 protein, we produced N and C terminal chimeric constructs between ChePoc1 and fluorescent proteins using the Gateway system (see Materials and Methods). Synthetic mRNAs were injected into eggs before fertilization, allowing localization of the tagged proteins to be followed in live developing embryos (Figure 3A). There were no obvious differences between results obtained using constructs with alternative fluorescent tags (Venus or mCherry), tags placed in N or C terminal position in the chimeras, or the inclusion of the endogenous 3′UTR to favor correct RNA localization and stability (not shown). A fluorescent signal was first detected around 5 hours post fertilization, at the beginning of the blastula stage. At this stage both Venus-ChePoc1 and ChePoc1-mCherry were visible in scattered cells of the developing embryo as sparse cytoplasmic aggregates (Figure 3Aa–f). Levels of cytoplasmic fluorescence were negligible, and no evidence was found for association with other cellular organelles including mitochondria, as demonstrated by comparison with the pattern of an endogenous, maternally-expressed GFP, which we fortuitously discovered to be naturally targeted to mitochondria. (Fourrage et al, manuscript in preparation). This GFP, CheGFP2, colocalizes with the mitochondrial indicator dye TMRE (Figure 3B), and its amino acid sequence contains an N-terminal mitochondrial targetting motif.

By 24 hours after fertilization, the injected embryos had developed into normal planula larvae with two cell layers, a well-defined ciliated ectoderm and a partially organized mass of presumptive endoderm cells. The tagged Poc1 proteins decorated the entire ectodermal cell layer with very regular array of bright dots (Figure 3Ag–i), the level of protein in the rest of the cytoplasm being almost undetectable. This pattern corresponds to that of the basal bodies positioned at the base of the cilium at the apical pole of each ectodermal epithelial cell. Scattered Poc1-mCherry dots were also detectable in the endodermal cells (data not shown). Poc1-mCherry colocalized perfectly in fixed embryos with immunofluorescence detection of the centrosome-enriched protein gamma-tubulin at the basal bodies in ectodermal cells, and also with gamma-tubulin dots at the spindle poles in dividing endodermal cells (Figure 3C). In the *Clytia* embryo, Poc1 thus localizes strongly both to centrioles in dividing cells and to the basal bodies of ciliated ectodermal cells. No significant Poc1 localization was detected elsewhere, including axonemes, microtubules or cytoplasmic organelles.

### Anti-HsPoc1 Stains Mitochondria But Does Not Recognize ChePoc1

**Figure 4:**
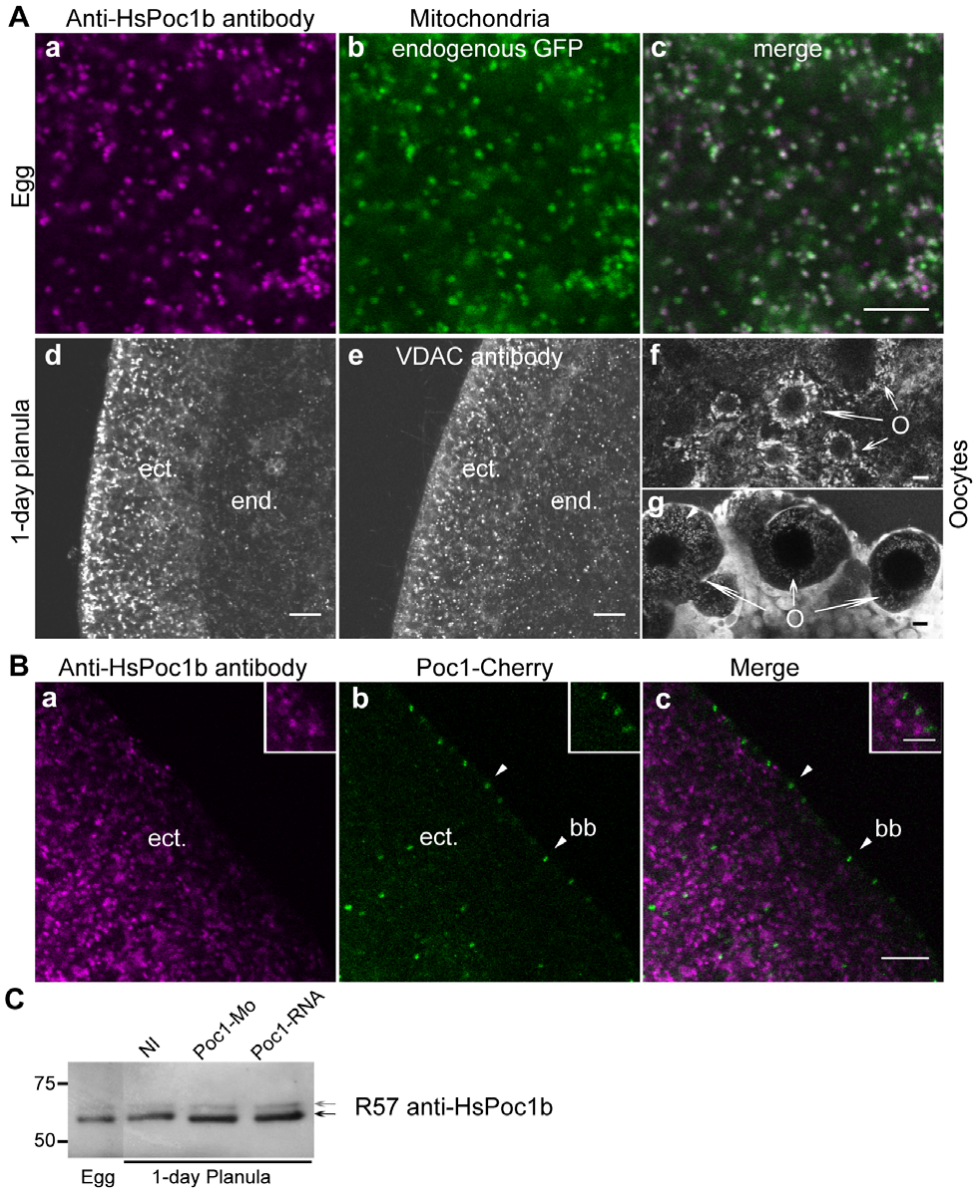
An Anti-Human Poc1 antibody recognizes mitochondria but not *Clytia* Poc1. A. HsPoc1 immunofluorescence (a,d,f) staining (mixed R56 and R57 antibodies) compared In spawned eggs (a–c), HsPoc1 co-localized with the mitochondria-targeted endogenous maternal GFP2 (b), while in larvae (d,e) it strongly resembled staining performed in parallel with rabit anti-VDAC antibody (e: ect. = ectoderm; end. = endoderm) and in oocytes (O) in live ovary pieces (f–g) with TMRE staining (g). Scale bars: 10 µm. B. HsPoc1 antibodies R56 and R57 (a) do not colocalize by immunofluorescence with Poc1-mCherry expressed protein (b) at ectodermal (ect) basal bodies (bb) in fixed planula larvae. In the merged images (c) the Poc1 antibody staining is shown in purple and Poc1-mCherry in green. Scale bar: 10 µm. Insets show enlarged basal bodies. Scale bar: 5 µm. C. Western blot probed with the Anti-HsPoc1b R57 antibody of groups of ten *Clytia* eggs or 1 with mitochondrial markers. 1-day planula larvae harvested after injection of 3 mM Poc1-MO and/or 1 µg/µl Poc1-RNA. Arrows indicate mayor and minor bands recognized by the antibody. The detected proteins are not depleted in the Poc1-MO extracts, nor enhanced in the ChePoc1 over-expressed extracts.

We attempted to address the localization of endogenous Poc1 in developing *Clytia* embryos by immunofluorescence using previously characterized affinity-purified rabbit anti-human Poc1 antibodies (R56 and R57, [8]). Unfortunately our experiments with these antibodies led us to conclude that they do not recognize *Clytia* Poc1. We document them briefly here (Figure 4), because as reported in human and *Xenopus* these antibodies strongly and specifically decorated mitochondria in *Clytia*. The possibility that this conserved mitochondrial staining is artefactual is important to consider for others in the field. The vesicular pattern observed in *Clytia* with the R56/R57 antibodies colocalized with the endogenous mitochondrial GFP present in eggs (Figure 4Aa–c), and closely resembled that obtained with another rabbit antibody recognizing the mitochondrial protein VDAC (staining of planula larvae shown in Figure 4Ad,e) as well as the vital dye TMRE (staining of ovary pieces shown in Figure 4Af,g, note the similarity of the ‘vermicelli’ structures in the oocytes). No basal body or centrosome staining was observed with the anti-HsPoc1 antibodies, as confirmed by immunofluorescence of fixed embryos pre-injected with Venus-Poc1 or Poc1-mCherry, in which the spots of fluorescence-tagged protein were clearly distinct from the strong mitochondrial localization of the R56 or R57 antibodies (Figure 4B). We cannot rule out the possibility that the antibodies recognize a second *Clytia* Pix protein with a distinct localization, however, this seems unlikely since only single Poc1 genes were identified in the two available fully sequenced cnidarian genomes (*Hydra* and *Nematostella*) and other non-vertebrate metazoan genomes that we surveyed. Furthermore, the major peptide (apparent Molecular Weight 60 KDa) recognized by each antibody in Western blot experiments in *Clytia* egg and embryo extracts, showed no reduction in intensity in Poc1-MO pre-injected planula-stage embryos compared to uninjected controls (Figure 4C), despite phenotypic abnormalities consistent with abrogation of centriole function (see below). This finding also precludes the possibility that the antibodies preferentially recognized a specific post-translationally modification or splice variant of the ChePoc1 protein targeted to mitochondria, not produced by the fluorescent-tagged RNA constructs.

Overall, our localization studies in *Clytia* provide evidence that ChePoc1 is targeted overwhelmingly to centrioles/basal bodies in developing embryos.

### ChePoc1 Depletion Disrupts Cell Division

To address ChePoc1 function, we attempted to deplete the protein in embryos by injecting a Morpholino antisense oligonucleotide (Poc1-MO) targeted to the predicted translation start site prior to fertilization to block translation. The specificity of the phenotypes observed was completely validated by ‘rescue’ experiments with the corresponding mRNA (see below), and supported by normal development of mock (buffer)-injected embryos (not shown). Note that Morpholino-based inhibition only affects protein synthesized after fertilization; it is likely that a stock of maternal protein unaffected by the morpholino is present until at least the blastula stage, with zygotically synthesized protein then progressively replacing the maternal pool. Major developmental events occurring following the blastula stage in *Clytia* include the onset of ciliogenesis in the ectoderm to mediate directed swimming, gastrulation by unicellular ingression from the future ‘oral’ pole to generate distinct ectodermal and endodermal cell layers, elongation of the embryo into a torpedo–shaped planula larva, and finally epithelialization of the initially disorganized endodermal cell mass [11].

**Figure 5:**
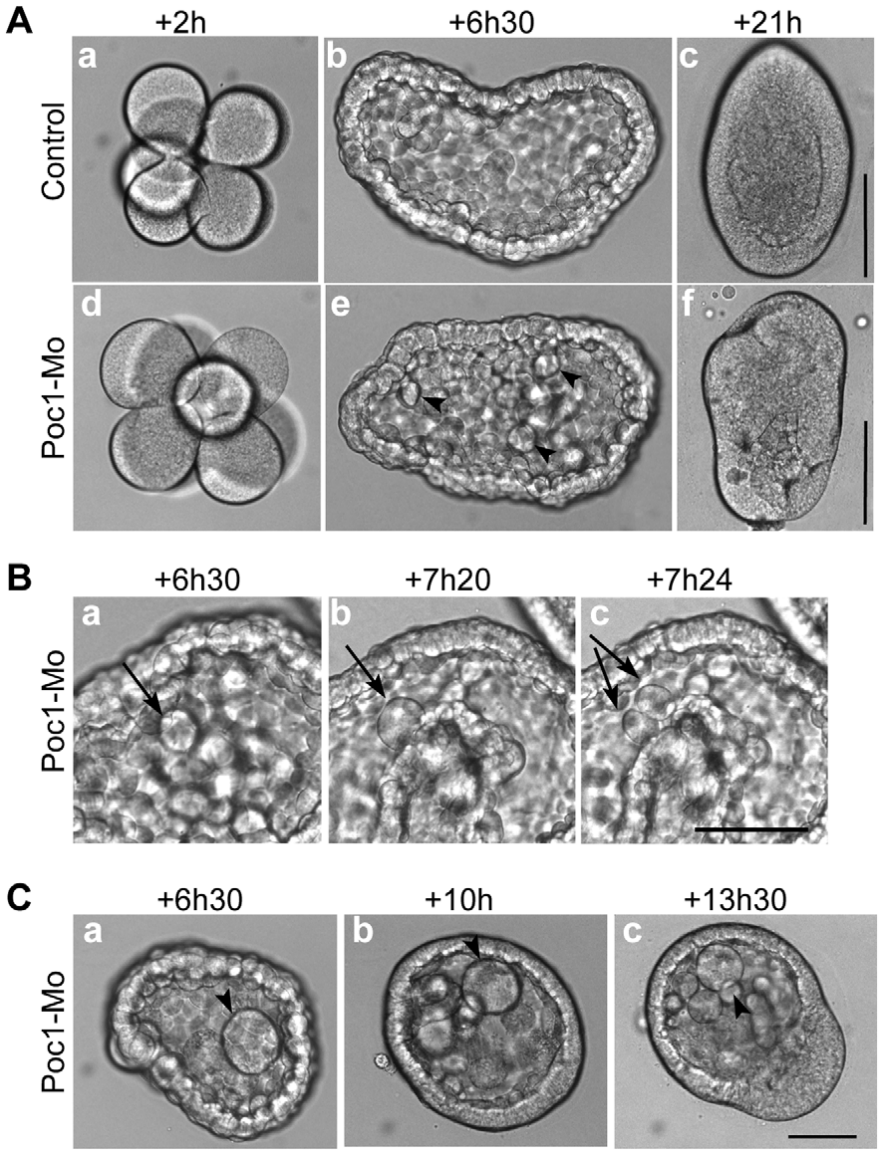
Poc1 MO disrupts cell division. Images from time-lapse recordings of *Clytia* embryos injected with Poc1-MO before fertilization (Movie S1). Times post fertilization are indicated. A. Cleavage divisions in Poc1-MO embryos proceeded in line with uninjected controls (a,d). Starting from around 6 hours post fertilization (blastula stage: b,e), Poc1-MO embryos showed lengthening of the cell cycle in some cells, followed by cell division defects/arrest, resulting in the appearance of larger cells (arrowheads). The accumulation of large cells in the blastocoel contributed to disruption and delay of gastrulation, resulting in characteristically deformed planula larvae (c,f). B. Cell cycle lengthening in a Poc1-MO blastula. Arrows identify a cell already larger than those around it cell which remains un-divided for at least 50 minutes (between a and b) before dividing (c). In uninjected controls filmed in parallel, cell division was largely synchronous until the blastula stage, with division occuring every 30 minutes (Movie S1, Figure 5A). C. Accumulation of abnormally large cells (arrowheads) in the blastocoel cavity (a), which remained undivided during gastrulation (b, c). Scale bars: 100 µm. Times indicated are hours post fertilization at 18°C.

Poc1-MO, injected as 2 or 3 mM solutions, resulted in phenotypes of varying severity by the planula stage, ranging from slight abnormalities in shape and swimming direction to aberrant spherical or irregular morphology, showing many abnormally large cells particularly in the endodermal region, and reduced swimming activity. To understand the origin of these defects we made time–lapse films of embryogenesis (Figure 5, Movie S1). Morpholino-injected embryos underwent cleavage divisions in parallel with uninjected controls until the early blastula stage, although with a loss of division synchrony due to cell cycle lengthening to a variable extent in different cells (Figure 5A, B). Progressively increased incidence of cell division delay or arrest was observed from the mid-blastula stage (5–7 hours post fertilization, with some individual cells detaching from the cell monolayer of the blastula either to be lost (eg in the right-hand embryo in Movie S1) or to accumulate in the blastocoel (Figure 5C). Subsequently the normal gastrulation process, which involves cellular ingression from the future oral pole and migration of internalized prospective endoderm cells along the inner blastocoel wall, was disorganized and delayed, although endoderm formation was eventually completed (Movie S2) with approx 1.5 hours delay. The final elongation of the planula was less pronounced than in control and the surface irregular (Figure 5Ac, f).

**Figure 6:**
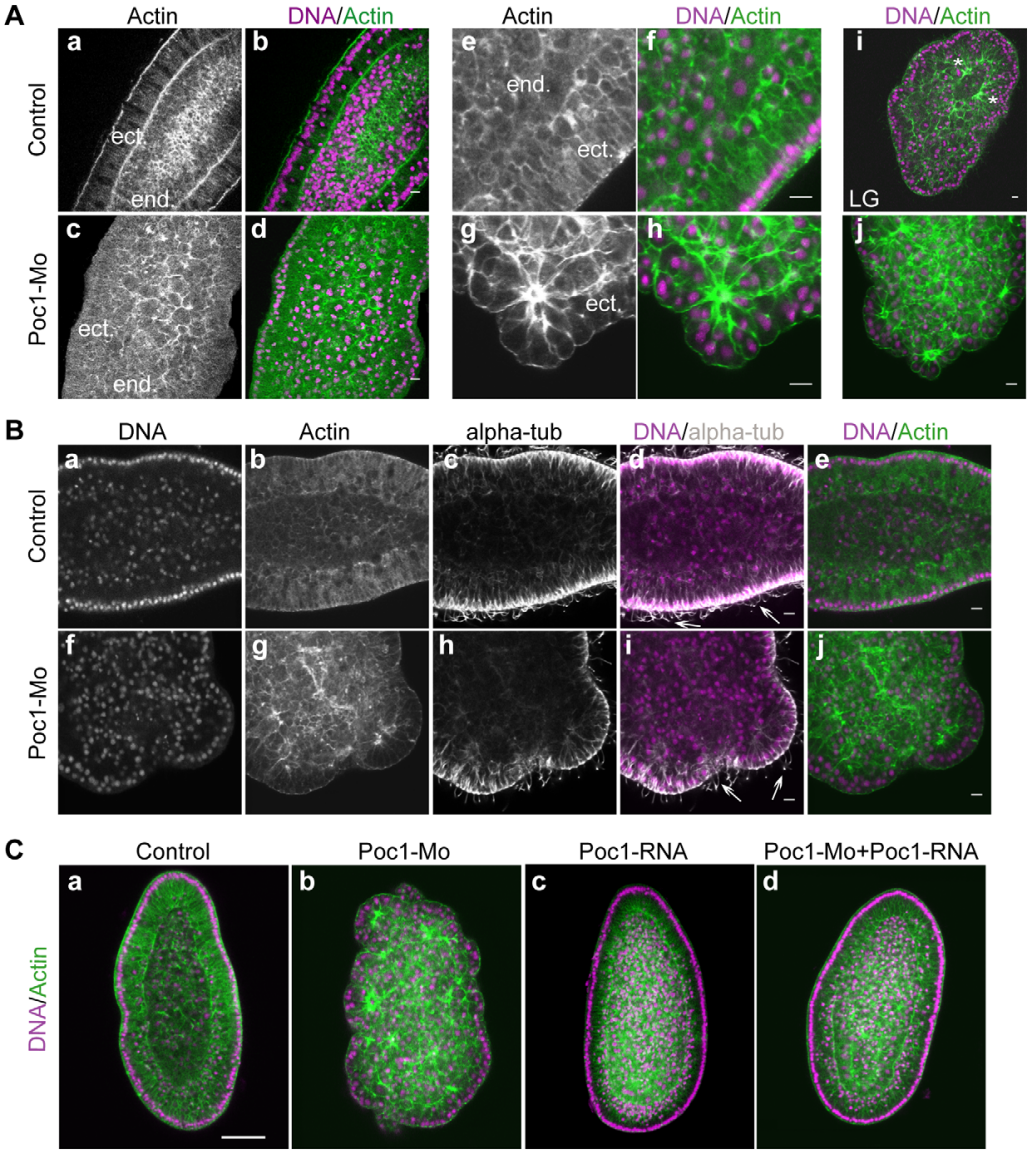
Poc1-MO disrupts gastrulation but not ciliogenesis. A. Confocal images of planula-stage embryos fixed one day after fertilization showing nuclear DNA labeled with Hoechst dye (purple in overlay) and actin with rhodamine-phalloidin (green in overlay). Poc1-MO at 2 mM (c,d) had little effect on the morphology of the ectoderm (ect), but cells in the endoderm were noticeably larger than in uninjected controls (a,b) and failed to organize into an epithelial layer. Following injection of Poc1-MO at 3 mM, the ectodermal layer was also found to be thickened compared to uninjected controls observed at the same time (e–f) and the cells organized in distinctive cell “rosettes” joined by strong basal actin bunches (g–h). The ectodermal organization in Poc1-MO embryos (h) closely resembled that of un-ingressed ectoderm cells (stars) in uninjected control embryos (g) at the late gastrula (LG) stage, consistent with the delay in gastrulation observed in time-lapse experiments. B. Confocal images of one-day planula–stage embryos stained with Hoechst dye (purple in overlay), rhodamine-phalloidin (green in overlay) and anti-alpha-tubulin to visualize microtubules including cilia (white in overlay, arrows). Pre-injection of 3 mM Poc1-MO (bottom row of images) caused severe disruption of morphology but did not prevent cilia growth on the ectodermal cells. C. Confocal images of one-day planula–stage embryos stained as in A, comparing uninjected embryos (a) with ones pre-injected with Poc1-MO (b), Poc1-mRNA alone (c) or Poc1-MO followed by Poc1-mRNA (d). The defects caused by Poc1-MO were abolished by subsequent mRNA injection, demonstrating specificity of phenotype. Poc1-mRNA alone had no major effect on cell or embryo morphology. Scale bar: 50 µm.

Detailed analysis of cell morphology in planulae fixed 24h after fertilization revealed that 2 mM Poc1-MO caused severe disorganization in the endodermal region with many cells and nuclei abnormally large (compare Figure 6Ac,d with control larva in Figure 6Aa,b), while the ectoderm was relatively unaffected. The enlarged cells and nuclei presumably result from prior cell cycle arrest or delay as observed in the time-lapse films. The characteristic central zone of fragmented nuclei present in 2 and 3-day planulae, associated with the formation of the endodermal cavity, was also enlarged in Poc1-MO embryos (not shown). When injected at 3 mM, Poc1-MO produced an even stronger phenotype, characterized by severe disorganization not only in the endodermal region but also of the ectodermal layer. These embryos had an irregular cauliflower shape, exhibiting multiple ectodermal folds and bulges, and associated swimming defects. At the cellular level the ectodermal bulges were found to contain striking “rosettes” of abnormally large ectodermal cells, their basal sides constricted together in association with accumulations of cortical actin (Figure 6Ag, h). Consistent with the marked delay in gastrulation provoked by the Poc1-MO, similar cauliflower-contoured embryos with actin knots at the base of the ectoderm were also seen in control embryos at the late gastrula stage (Figure 6Ai). These structures appear to reflect, at least in part, deformation of the ectoderm by traction of underlying migrating endodermal cells during gastrulation.

**Figure 7:**
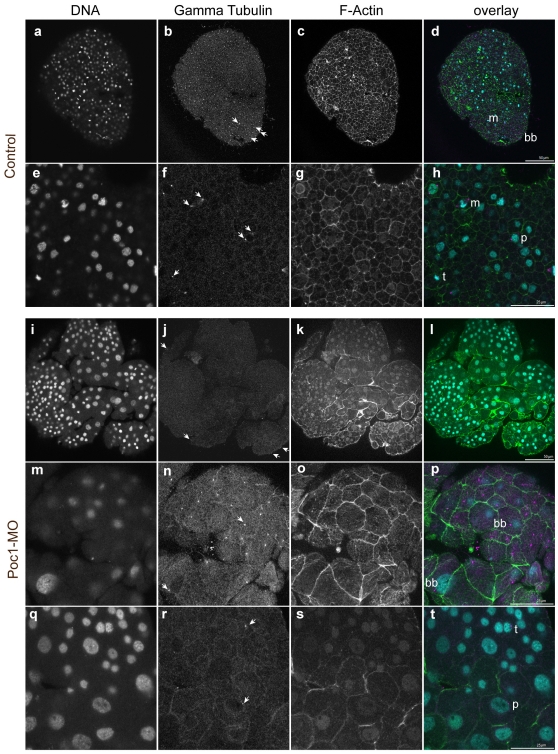
Centrosomes in Poc1-MO embryos. Confocal images of early gastrula-stage embryos stained with Hoechst dye for DNA (a, e, i, m, q), anti-gamma tubulin immunofluorescence (b, f, j, n, r) and Alexa-488-phalloidin for cortically-enriched F actin. In the overlays (d, h, l, p, t) DNA is blue, gamma tubulin purple and F-actin green. Control uninjected embryos (a–h) showed bright gamma tubulin dots (arrows) at the basal bodies of ectodermal cells (bb) and centrosomes of cells undergoing mitosis (prophase: p; metaphase: m; telophase: t). In embryos derived from Poc1-MO eggs (i–t), the size of the cells and the shape of many nuclei were irregular, indicating cell cycle disruption, however regular basal bodies (bb) remained clearly detectable in confocal sections through the apical ectoderm (m–p), and cells undergoing normal mitosis each with two gamma tubulin-rich centrosomes were detectable in deeper confocal planes (q–t).

Poc-1-MO embryos and control uninjected embryos were fixed in parallel at the early gastrula stage and stained with anti gamma tubulin antibodies to address centrosomal behaviour (Figure 7). In control embryos, bright dots of gamma tubulin staining decorated the basal bodies in each ectodermal cell, and centrosomes through mitotic stages from prophase to telophase. During interphase, centrosomes were undistinguishable above the background of cytoplasmic/perinuclear gamma tubulin protein. In the Poc1-MO, many cells were abnormally large, consistent with the cell cycle lengthening seen in the video analysis; however, nuclear morphology and gamma-tubulin staining of mitotic figures and basal bodies was indistinguishable from controls. Normal nuclei, gamma-tubulin stained centrosomes and basal bodies were also detected in embryos fixed at later (planula) stages, along with many abnormal/degenerating nuclei lacking gamma tubulin-stained centrosomes (not shown). Intrerpretation of the abnormalities seen in planula larvae is hard to assess, because nuclear degeneration was also observed in the central region of control planula at this stage, as a part of endodermal cavity morphogenesis. The absence of abnormal spindles in the enlarged cells of Poc1-MO embryos, suggests that mitosis occurs correctly but less frequently when Poc1 protein levels are diminished. If Poc1 plays the same centriole biogenesis role as demonstrated in other systems, this could reflect the operation of a cell cycle checkpoint in the absence of two fully functional centrioles [17].

Although Poc1-MO injected embryos showed severely deformed morphology, they began to swim at the same time as controls (Movie S2). To assess whether ciliogenesis was affected by Poc1 depletion, we performed anti-alpha tubulin immunofluorescence on Poc1-MO injected and control embryos fixed at 24 h post fertilization (Figure 6B). Despite the clear disruption to overall morphology, abundant cilia were detected on the epithelial surface (Arrows in Figure 6Bd, i). Thus, ciliogenesis in *Clytia* either does not require Poc1, or can be supported by reserves of maternal protein. This latter possibility is quite plausible, and indeed the likely persistence of maternal Poc1 protein means that all the Poc1-MO phenotypes must be interpreted with caution, as reflecting only partial and progressive depletion of the protein.

To verify that the phenotype of Poc1-MO embryos could be specifically ascribed to (partial) Poc1 protein depletion, we injected mRNA coding for the ChePoc1ORF but lacking the Morpholino target site into eggs previously injected with Poc1-MO (Figure 6C). The severity of morphological disruption morphology was scored according to an arbitrary index with 0 being normal and 4 being severely disrupted (Table 1). While all embryos pre-injected only with Poc1-mRNA were morphologically indistinguishable from uninjected controls (compare Figure 6Ca with c), Poc1-MO embryos showed phenotypes of index 2 to 4. Subsequent injection of Poc1 mRNA restored a near normal development (Figure 6Cd; index values 0 or 1).

**Table 1:** Rescue of Poc1-MO phenotype by Poc1-mRNA.

Poc1-MO phenotype	Control	Poc1-MO	Poc1-mRNA	Poc1-MO+Poc1-mRNA
**Index 0: WT**	30 (100%)	-	8 (100%)	10 (47%)
**Index 1: slightly irregular shape**	-	-	-	10 (47%)
**Index 2: large cells in endoderm, 1–2 folds in ectoderm**	-	6 (33%)	-	1 (5%)
**Index 3: large cells in endoderm and ectoderm, several folds in ectoderm**	-	6 (33%)	-	-
**Index 4: large cells in ectoderm, absence of real endoderm, numerous folds in ectoderm**	-	6 (33%)	-	-
**Total**	**30**	**18**	**8**	**21**

Taken together the Morpholino injection experiments strongly suggest that zygotically synthesized Poc1 protein helps ensure the normal progression of cell division in *Clytia* embryos as the store of maternal protein is depleted. The greater sensitivity of the endoderm to Poc1 reduction probably reflects the earlier differentiation of the ectoderm layer, associated with a reduction of the frequency of cell division. All of the phenotypic features of Poc1-MO embryos can be attributed to the effects on cell division, progressive accumulation of undivided or slowly dividing cells being the probable cause of the gastrulation defects.

## Discussion

Understanding the centriole has long been a major challenge in cell biology, since this complex organelle is essential in its basal body form for the genesis and function of cilia and flagella across the eukaryotic world, and, as a cytoplasmic structure, can also be a key player in cell division [18]. The increasing awareness that ciliogenesis defects underlie many human pathologies has further increased the desire to fully characterize the structure and function of these fascinating organelles [19]. In this study we provide evidence that the highly conserved protein Poc1, previously proposed to have both centriolar and non-centriolar roles, is localized exclusively to centrioles in the cnidarian *Clytia* and that its function there is likely also evolutionary conserved [8]. These observations further reinforce the picture of a highly conserved and uniquely centriolar localization and function for Poc1 across eukaryotic organisms.

### Poc1 is a Highly Conserved Centriole Protein

Tagged fluorescent Poc1 proteins expressed in *Clytia* embryos proved to be excellent in vivo centriole markers, with bright staining confined to cytoplasmic centrosome and basal bodies. Negligible fluorescence was detected elsewhere in the cell, indicative of significant protein turnover in the cytoplasm. In line with this strict localization to the centriole, embryos pre-injected with Poc1-MO to prevent neosynthesis of the protein following fertilization showed slowing/arrest of cell division, the accumulation of undivided cells likely responsible for the observed gastrulation delay and morphological defects. Given the presence of maternal protein, the observed effects of the morpholino are due to a partial and progressive reduction of Poc1 protein levels rather than to complete loss of function. Although this experimental system is thus not ideal to analyze the precise role of Poc1 at the centrosome, the observed cell division defects are entirely consistent with the various phenotypes documented previously following interference with Poc1 protein levels in a range of other species and cells. These defects, relating to different aspects of centriole biology (duplication, elongation and ciliogenesis), can all be ascribed to the participation of Poc1 in the structure and integrity of centrioles and basal body, with a particular importance at the time and site of daughter centriole formation [6]–[8], [20], consistent with its ultrastructural localization.

The hypothesis that Poc1 has an evolutionary conserved and essential function is supported by the remarkable sequence similarity between Poc1 proteins in diverse eukaryotic species. In this context, it is somewhat surprising that the Poc1 gene is absent from the genomes of some species, including the nematode *Caenorhabditis* amongst the metazoans, angiosperms and most but not all fungi [1]. The implied sporadic losses of Poc1 during evolution may have occurred after its function was adopted by another protein, and/or in cases where centrioles are less vulnerable to mechanical stress. The latter idea is consistent with the observed correlation between the presence of Poc1 and that of beating cilia [1]. Incidentally our results do not support the hypothesis of a specific role in ciliogenesis for Poc1 in *Clytia*, as demonstrated in zebrafish and human epithelial cells [7]; Poc1-Mo embryos showed severe division and morphology defects without apparently affecting ciliation of the ectoderm cells at the onset of gastrulation. We cannot rule out, however, the possibility that inherited maternally derived protein remaining at this stage is sufficient to support ciliogenesis, with centriole duplication in dividing cells showing a greater sensitivity to reduced Poc1 levels. The persistence of significant levels of maternally–derived Poc1 protein to the early gastrula stage can also explain why less disruption was observed in the ectodermal layer, where epithelial differentiation starts around this time, than in the endodermal region, where active division cycles continue for many more hours before epithelialization.

### Poc1 is Exclusively Centriolar

Given previous reports of a germ plasm localization for Poc1 protein in *Xenopus* oocytes [8], we were initially excited by the observation of a punctate perinuclear distribution of the *Clytia* Poc1 mRNA in pre-vitellogenic oocytes, a site rich in mitochondria where germ plasm mRNAs are also localized. This perinuclear mRNA localization requires further investigation, to determine whether it involves true mitochondrial targeting or simply non-specific accumulation in a region concentrated in many maternal mRNAs. Unlike the reports in *Xenopus* oocytes and in cultured vertebrate cells, we found no hint of Poc1 protein localization to mitochondria or germ plasm in *Clytia*, the targeting of fluorescent proteins appearing exclusively centriolar. The relationship of Poc1 with mitochondria merits reinvestigation in vertebrates in the light of our conclusion in *Clytia* that anti-Human Poc1 antibodies recognize mitochondria but not the Poc1 protein [8]. Whether or not Poc1 localization to mitochondria/germ plasm is confirmed, it will not challenge the vision of this protein as an integral centriolar protein, highly conserved in sequence, localization and function since the beginning of eukaryotic evolution.

## Materials and Methods

### Identification and Phylogenetic Analysis of *Clytia* Poc1

A *Clytia hemisphaerica* cDNA clone containing the complete ORF of the *Clytia* POC1 gene in Express-1 vector was retrieved from a mixed-stage cDNA library following identification from our EST collection, sequenced by the Genoscope (Evry, France). GenBank accession number: ChePOC1: HM010924.

For phylogenetic analysis, Poc1 amino acid sequences were retrieved by BLAST from publicly available protein datasets (Table S1). The selected sequences were aligned together and with an out-group comprising sequences of the related WD40 domain protein Lis1, using CLUSTALW in BioEdit and corrected by eye (Supplementary Figures S1 and S2). Maximum Likelyhood analysis was performed on the WD40 domain + POC1 domain using PhyML, with conditions exactly as described previously [15].

### ChePoc1-Fluorescent Protein Chimeras

N-terminal or C-terminal fluorescent protein chimeras were constructed using Gateway technology [21]. The ChePOC1 ORF was amplified by PCR and cloned into TOPO plasmid using the pENTR/D TOPO kit (Invitrogen) and then into pSPE3 destination vectors, kindly provided by Céline Hébras, to create the following 5 constructs: Venus-Poc1ORF; Venus-Poc1ORF-3′UTR; Poc1ORF-Venus; Poc1ORF-mCherry; Poc1ORF alone. mRNA for microinjection was transcribed from linearized plasmids using the mMessage mMachine kit (Ambion), poly(A) tailed using the Poly(A) Tailing kit (Ambion) and resuspended in H_2_0 for injection into eggs at 0.5 µg/µl to 1 µg/µl prior to fertilization.

### Manipulation of *Clytia* Eggs and Embryos

Eggs and embryos were obtained from adult *Clytia hemisphaerica* medusae, derived from permanent laboratory colonies [22]. Spawned eggs were microinjected using a Femtojet apparatus (Eppendorf) prior to fertilization as previously described [23]. An antisense morpholino oligonucleotide (GeneTools) targeted to the putative AUG initiation codons of ChePoc1 was injected at concentrations of 2 or 3 mM: Poc1-MO, 5′-CACAUUUUCACUUAUUCACUAAUUC-3′. For rescue experiments, eggs we re first injected with the Poc1-MO, and then a sub-group of these eggs re-injected with synthetic Poc1ORF mRNA lacking the MO binding sequence, prior to fertilization.

### Microscopy

In situ hybridization [22] and immunofluorescence staining [13] were performed as described previously. Primary antibodies: mouse monoclonal anti-alpha tubulin DMIA and rabbit anti-gamma tubulin GTU-88 (Sigma), rabbit anti-VDAC (gift of M. Colombini) [10]; affinity purified rabbit polyclonal antibodies R56 and R57 recognizing human Poc1a and Poc1b (generous gift of A. Fry) [8]. Nuclei were stained with Hoechst dye 33348 and the actin-rich cortical outlines of the cells with rhodamine-phalloidin or Alexa-488-phalloidin [13]. Mitochondria were labeled in live cells by incubation for 5–30 minutes in 0.1 mM TMRE (Tetra-methylrhodamine ethyl ester; Invitrogen) in 0.2 µm Millipore filtered seawater, diluted immediately prior to use from a 1 mM stock in ethanol. Fluorescent specimens were imaged using Leica SP2 or SP5 Confocal microscopes. Time lapse films using bright field illumination were recorded on a Zeiss Axiovert inverted microscope equipped with motorized stage driven by Metamorph software. For films from the onset of ciliogenesis at the late blastula stage, embryos were embedded in 0,3% w/v Low Melting Point agarose in 0.2 µm Millipore Filtered Sea Water. The solidified agar prevents the embryos from swimming out of the observation field, but does not prevent them from rotating around their long axis.

### Western Blotting

Samples were processed for Western blotting as described previously [15], [24], using anti-human Poc1 antibodies R56 and R57 [8].

## Supporting Information

Table S1 POC1 gene orthologues in eukaryotic genomes.
(0.04 MB DOC)

Figure S1 Full Alignment of all Poc1 amino acid sequences used for phylogenetic analysis. (Black boxes: 100% sequence similarity. Background shading indinxcates the WD40 and Poc1 domains.
(9.97 MB TIF)

Figure S2 Amino acid alignment of WD40 and Poc1 domains used for the phylogenetic analysis shown in Figure 1. (Black boxes: 100% sequence similarity).
(1.05 MB TIF)

Movie S1 Comparison of cleavage divisions in an uninjected embryo (left) and two derived from eggs injected with 2mM Poc1-MO (center and right) filmed in parallel. The sequence covers 3 hours from the 8-cell to the mid blastula stage, with images acquired using DIC optics every 4 minutes. Cells in the center embryo continue to divide during the sequence, although a slight slowing and desynchronization of the regular division cycles in observed. The right embryo shows a stronger effect with some cells showing a marked division delay and detaching from the embryo.
(2.83 MB MOV)

Movie S2 Comparison of gastrulation in an uninjected embryo (left) and one developed from an egg injected with 2mM Poc1-MO (right) filmed in parallel. The sequence covers 19 hours with images acquired using DIC optics every 4 minutes. The embryos were filmed in agarose wells to retain them in the observation field, but continue to swim and rotate around the embryonic polarity axis by ciliary beating. Gastrulation proceeds by cell ingression from the oral pole (top) in both embryos, but is much delayed and more irregular in the Poc1-MO embryo. Note that some undivided cells are already present in the blastocoel of the Poc1-MO embryo prior to gastrulation, and further accumulate during the period of the film, likely contributing to the gastrulation difficulties.
(17.24 MB MOV)
