# Supplementary material for: A Highly Conserved Poc1 Protein Characterized in Embryos of the Hydrozoan Clytia hemisphaerica: Localization and Functional Studies
Source: PLoS One. 2010 Nov 16;5(11):e13994. doi: 10.1371/journal.pone.0013994 (PMC2982836; doi:10.1371/journal.pone.0013994)
Supplement: Table S1 — POC1 gene orthologues in eukaryotic genomes. (0.04 MB DOC) [file pone.0013994.s001.doc]

**Table S1. POC1 gene orthologues in eukaryotic genomes.**

| **Species** | **BLAST** | **Accession number** |
| --- | --- | --- |
| *Clytia hemisphaerica* | EST | HM010924 |
| *Hydra magnipapillata* | Genome Compagen | 162437 |
| *Nematostella vectensis* | Genome JGI | contig 38964 |
| *Capitella species* | Genome JGI | 149802 |
| *Branchiostoma floridae* | Genome JGI | 205723 |
| *Homo sapiens* | NCBI | NP_758440.1 (HsPoc1b) and NP_056241.2 (HsPoc1a) |
| *Dario rerio* | NCBI | XP_702605 (DrPoc1a) and XP_702204 (DrPoc1b) |
| *Xenopus laevis* | NCBI | NP_001089468.1 (XlPoc1b) and NP_001079883.1 (XlPoc1a) |
| *Lottia gigantea* | Genome JGI | 237317 |
| *Ciona intestinalis* | Genome JGI | XP_002127877.1 |
| *Monosiga brevicollis* | NCBI | XP_001743496.1 |
| *Chlamydomonas reinarhdtii* | Genome JGI | XP_001699653 |
| *Physcomitrella patens* | Genome JGI | 133767 |
| *Batrachochytrium dendrobatidis* | Genome JGI | 86053 |
| *Tetrahymena thermophila* | NCBI | XP_001029778 |
| *Paramecium tetraurelia* | Genome JGI | CAK94781.1 |
