# Supplementary figures and images for: A Highly Conserved Poc1 Protein Characterized in Embryos of the Hydrozoan Clytia hemisphaerica: Localization and Functional Studies
Source: PLoS One. 2010 Nov 16;5(11):e13994. doi: 10.1371/journal.pone.0013994 (PMC2982836; doi:10.1371/journal.pone.0013994)

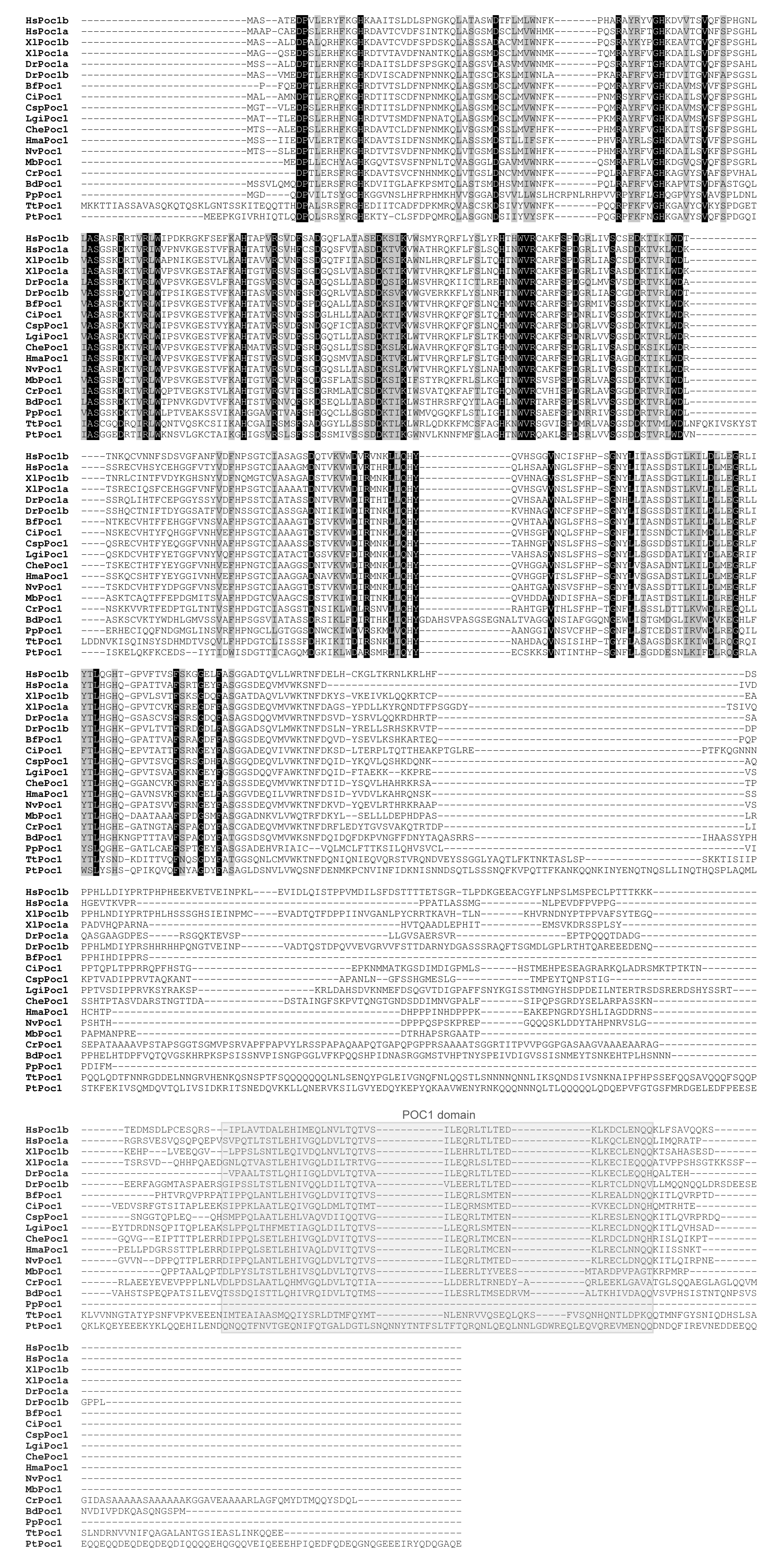

Supplement: Figure S1 — Full Alignment of all Poc1 amino acid sequences used for phylogenetic analysis. (Black boxes: 100% sequence similarity. Background shading indinxcates the WD40 and Poc1 domains. (9.97 MB TIF) [file pone.0013994.s002.tif]

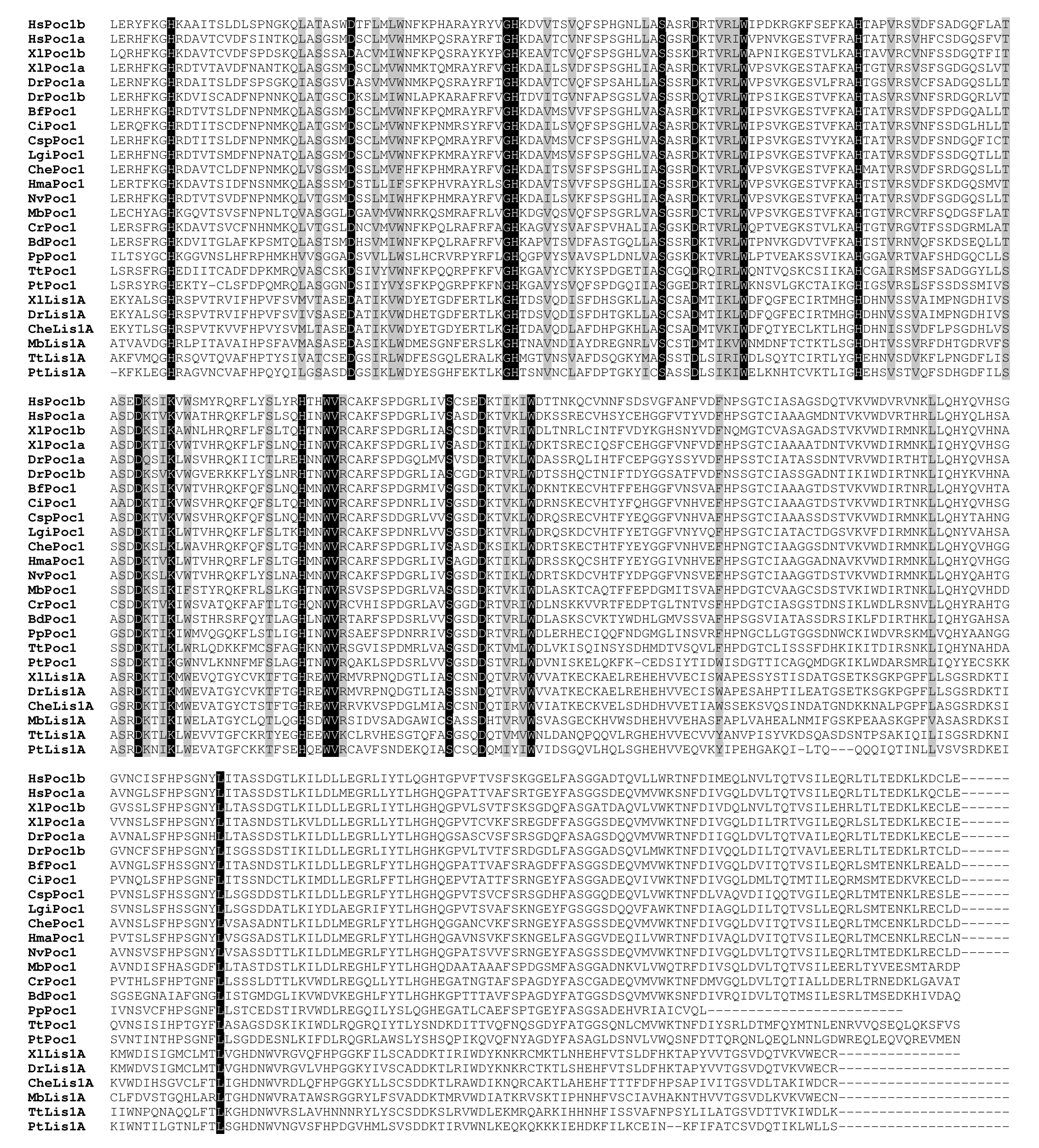

Supplement: Figure S2 — Amino acid alignment of WD40 and Poc1 domains used for the phylogenetic analysis shown in Figure 1. (Black boxes: 100% sequence similarity). (1.05 MB TIF) [file pone.0013994.s003.tif]
